# Research on patients' willingness to conduct online health consultation from the perspective of web trust model

**DOI:** 10.3389/fpubh.2022.963522

**Published:** 2022-09-09

**Authors:** Yongxun Xu, Zishuo Yang, Hongyan Jiang, Peizhen Sun

**Affiliations:** ^1^School of Business and Management, Jilin University, Changchun, China; ^2^School of Business Administration, Xuzhou Vocational College of Industrial Technology, Xuzhou, China; ^3^School of Education Science, Jiangsu Normal University, Xuzhou, China; ^4^School of Economics and Management, China University of Mining and Technology, Xuzhou, China

**Keywords:** cognition-based information, affect-based information, institution-based information, online trust, health consciousness, willingness to conduct online health consultation

## Abstract

**Background and aims:**

The online health platform becomes an important choice for users to receive health services. While bringing convenience to users, it also provides lots of overloaded information for users and leads them to have trouble in making online medical choice decisions. In order to understand what types of information on the online health platform play key roles in the user's decision choice, this research explores the effects of cognition-based information, affect-based information and institution-based information on patients' willingness to conduct online health consultation from the perspective of Web Trust Model.

**Methods:**

Responses of 412 valid questionnaires were collected *via* online surveys.

**Results:**

The results showed that: (1) cognition-based information, affect-based information and institution-based information positively predict patients' willingness to conduct online health consultation; (2) online trust significantly mediates the relationship between online health platform information and willingness to conduct online health consultation; (3) health consciousness significantly moderates the mediating effect of online trust in the effect of online health platform information on patients' willingness to conduct online health consultation.

**Conclusions:**

The findings make theoretical contributions by extending the Web Trust Model to the research field of online health service and offers practical implications for how to effectively provide information on the online health platform.

## Introduction

With the advancement of modern technologies and people's increasing demand for health services (1), healthcare industry is stepping into digitalization transformation (2). Under this background, “Internet + health care” has become a hot topic of common concern to the government and academia. Along with the digitalization transformation of healthcare industry, the online health platform has become more and more popular.

The online health platform has both positive and negative effects. On the one hand, it provides users with health information and services through the Internet, which can improve the efficiency of medical services and reduce time costs (3). Accordingly, the online health platform becomes an important choice for users to receive health consultation services. Especially during the COVID-19 epidemic, due to the shortage of medical supplies in society and the inconvenience of people going out, the online health platform is more welcomed and favored by a large number of users based on its convenience (4). On the other hand, the online health platform may also cause information load to a certain extent, while bringing convenience to people. Compared to the traditional medical pattern, the online health platform can provide a large amount of overloaded information for users, which may lead users to have trouble in making online medical choice decisions. Therefore, the content of information provided by the online health platform plays a key role in the user's decision choice.

Previous studies have investigated the relationship between online health platform information and patients' choice (5–7). However, most of prior research has just focused on the single-dimensional information and relatively neglected multi-dimensional information on the online health platform. Specifically, scant academic research has simultaneously explored the effects of cognition-based, affect-based and institutional-based information on patients' willingness to carry out online health consultation. In addition, previous studies have mostly adopted crawler technology to obtain online health platform information directly from the website, which only revealed the phenomenon without deeply uncovering its underlying mechanism. As we known, scant academic research empirically addressed the inner mechanism underlying the effect of online health platform information from the perspective of patients' psychological perceptions. To fill this research gap, the current study aims to explore the effects of multiple types of online health platform information on patients' willingness to conduct online health consultation and its internal mechanism from the theoretical perspective of Web Trust Model (WTM).

Based on the background of medical digitalization transformation and people's urgent demand for online healthcare during the COVID-19 epidemic, it is of great significance to explore the factors influencing people's willingness to do online health consultation. Moreover, the Web Trust Model point out the direction for this research and lay a sufficient theoretical foundation for investigating the antecedents of the willingness to conduct online health consultation and its internal mechanism. The conclusion of this research can provide critical reference not only for elevating people's willingness to carry out online health consultation, but also for promoting the application and development of online health platform.

Overall, this paper is aimed to address three questions. First, we investigate whether the three dimensions (i.e., cognitive, affective, and institutional) of online health platform information exert significant influence on patients' willingness to carry out online health consultation. Second, by introducing online trust, the current study examines how online health platform information impacts patients' willingness to participate in online health consultation. Third, this study further explores the boundary condition for the indirect effect of online health platform information on the willingness to online health consultation *via* online trust. Accordingly, the results of this study could add new proof to the existing Web Trust Model (WTM) and extend it to the research field of online health services. Meanwhile, the findings also offer practical implications for how to effectively provide information on the online health platform.

## Theoretical background and hypotheses

**Figure 1:**
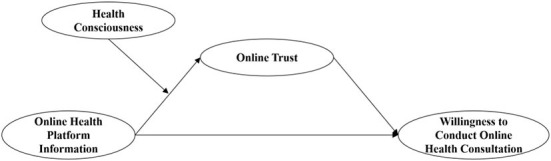
Conceptual research model.

The purpose of this study is mainly to test the effect of online health platform information on patients' willingness to carry out online health consultation, and the mediating role of online trust. Furthermore, health consciousness is identified as a key contingent factor in moderating the effect of online health platform information on online trust, and further leading to patients' willingness to conduct online health consultation. The conceptual research model is illustrated in Figure 1.

### The online health platform information and the willingness to conduct online health consultation

Online health platform provides convenience for physician-patient interaction, that is, individuals can receive health consultation services without leaving home. By reviewing and summarizing the related literature (5, 6, 8), this study finds that different from the traditional medical pattern, the online health platform information obtained by patients is more diverse. It can be divided into cognition-based information (i.e., physicians' personal quality), affect-based information (i.e., patients' affective expression to physicians), and institution-based information (i.e., information related to the operation of third-party platforms).

The Medium System Dependency Theory provides support for the relationship between online health platform information and the willingness to conduct online health consultation (9). The theory suggests that individuals' behaviors may change due to having access to information resources provided by the internet, that is, the intention of purchasing services may be improved when the utilization and dependence on online information resources is high (10). According to this theory, if the online medical platform can provide a large number of helpful information resources for users to adopt and rely on, they are more willing to choose online health consulting services. Previous research has also pointed out that consumers' online shopping intention tends to largely depend on online shopping information (11). Similarly, we infer that patients are the receivers of the service in the field of online healthcare, and they can analyze and compare the information on the online health platform before choosing online health consultation services. Therefore, online health platform information may play a crucial role in affecting patients' willingness to carry out online health consultation.

#### Cognition-based information and patients' willingness to conduct online health consultation

Cognition-based information refers to some information about physicians' personal quality. And competence and benevolence are very important elements of physicians' personal quality (12). Competence is defined as the related skills or professional characteristics possessed by physicians, which makes them professional in the medical field (6). While benevolence means that the extent to which physicians are patient-centered to help solve patients' health problems as much as possible, rather than for their own profits (13, 14).

Prior studies have shown that physicians' competence and benevolence are both important references for patients to choose the physician (5, 15). Physicians' competence is a key factor in determining whether they can provide high-quality health consultation to patients (16), and is also an important criterion for patients to choose whether to conduct online health consultation. Thus, if patients can perceive physicians' competence through physicians' cognition-based information on online health platform, indicating the professional skills and characteristics of physicians, they are more willing to do online health consultation. Similarly, physicians' benevolence is also a positive driver of patients' selection, which is indicated by the degree of physicians' effort (17). The more services the physician is obliged to provide (i.e., the articles voluntarily published by the physician), the greater efforts they put in. When perceiving the physicians' efforts in the online health service, patients are more likely to choose the online health consultation (5). To sum up, cognitive information about physicians' competence and benevolence obtained by patients on the online health platform may affect their choice of conducting online health consultation. Therefore, we propose the following hypothesis:

Hypothesis 1 (H1). The cognition-based information can significantly affect patients' willingness to conduct online health consultation.

#### Affect-based information and patients' willingness to conduct online health consultation

Affect-based information is defined as the certain type of information about physicians' online reputation reported by patients, including online ratings, the number of gifts, and overall satisfaction. On the online health platform, all patients belong to the same community, which are the receivers and users of online medical services. Moreover, the information generated by similar patients may become the basis for other patients to make decision choices (15). And some researchers have pointed out that online ratings and reviews provided by online platforms can reduce the social distance between service providers and users, thereby driving service users to choose (18, 19). Thus, we assume that affect-based information (e.g., online ratings or reviews related to physicians) on the online health platform can help patients choose to conduct online health consultation. Based on the above analysis, we hypothesize:

Hypothesis 2 (H2). The affect-based information can significantly affect patients' willingness to conduct online health consultation.

#### Institution-based information and patients' willingness to conduct online health consultation

Institution-based information is conceptualized as some structured guarantees for physician-patient interactions provided by the online health platform, including both initial accreditation and subsequent monitoring (20). In daily life, many consumers may choose to spend on online platforms that offer guarantees for them. Because of the guarantee of the platform, the risks of the transaction can be greatly reduced. Especially on the online health platform, patients are worried about their health information being leaked (21). Once privacy risks exist in health information, patients' willingness to conduct online health consultation can be seriously attenuated (22). Previous research has pointed out that review and authentication mechanism, privacy assurance mechanism, and dispute resolution mechanism can improve consumers' willingness to participate in sharing economy platforms (23), and then facilitate the online transactions (24). We thus predict that if the online health platform can provide patients with institution-based information, including the screening and review of physicians, the protection of patients' health information and the guarantee of carrying out health consultation services, patients are more willing to participate in online health consultation. Accordingly, we propose:

Hypothesis 3 (H3). The institution-based information can significantly affect patients' willingness to conduct online health consultation.

### The mediating role of online trust

Online trust is defined as the truster's belief that the trustee has attributes beneficial to the truster (25). Accordingly, in the context of online health platform, online trust is specific to the patient's belief that the online health platform provides many benefits to them. The Web Trust Model (WTM) (25) states that some factors may influence the individual's willingness and behaviors through the indirect effect of online trust. Among them, the antecedents of online trust include cognitive factors, affective factors (26), and institutional factors (25). Drawing on the Web Trust Model (WTM) (25), we infer that the cognition-based, affect-based and institution-based information provided by online health platform enable patients to build more trust in the online health platform. Then if patients think the online health platform is more trustworthy, they can exhibit higher willingness to carry out online health consultation.

In addition, some empirical studies have provided supports for the relationships between online health platform information, online trust and the willingness to do online health consultation. On one hand, previous studies have directly shown the significant relationship between the online information and patients' trust in physicians (27). On the other hand, studies have revealed that online trust was a driving factor influencing online purchase (28), which provided supports for the relationship between online trust and the willingness to do online health consultation. According to the analysis above, we conject that the online health platform information may significantly predict patients' online trust, and further impact their willingness to participate in online health consultation. Hence, we propose the hypothesis:

Hypothesis 4 (H4). Online trust can mediate the relationship between the online health platform information and patients' willingness to conduct online health consultation.

### The moderating role of health consciousness

Health consciousness is conceptualized as a psychological construction, which corresponds to one's concern for individual's health and the willingness to change behaviors to improve personal health (29, 30). Health consciousness is a pre-requisite for the trust building between physicians and patients (30). In other words, higher health-conscious patients are more likely to pay attention on the information provided by online health platform, and the patients' increasing attention to online information corresponds with their higher trust in physicians (31). When patients paid more attention on online health platform information, this information would help them better understand and believe the benefits that the online heath consultation could offer. In turn, increased online trust can make patients more willing to participate in online health consultation (25). Thus, online health platform information could trigger higher online trust for individuals with high health consciousness than those with low health consciousness, and then increase their willingness to conduct online health consultation.

To be specific, individuals with high health consciousness have greater ability to understand and evaluate the online health platform information (29), and then generate more optimistic expectations for physicians' ability and kindness. Thus, the online health platform information can help patients build higher trust (32, 33), which further elevate patients' willingness to do online health consultation. On the contrary, individuals with low health consciousness pay less attention to the online health platform information, and are less able to hold a positive expectation of the physician's online medical service, thus building lower online trust. And lower online trust may reduce patients' willingness to accept online health consultation. Therefore, the effect of the online health platform information on online trust may be strengthened when health consciousness is high (vs. low). Additionally, previous research has found that online trust exert a positive effect on the willingness to do online health consultation (25). Based on Hypothesis 4 and the above analysis, the following hypothesis is formulated:

Hypothesis 5 (H5). Health consciousness moderates the effect of the online health platform information on patients' willingness to conduct online health consultation *via* online trust.

## Methodology

### Data collection

We first got the approval of the Institutional Review Board at the University of the first author. In this study, a random sampling method was adopted to investigate the users of the online health platform. Informed consents were provided by all the participants. All questionnaires in this study were anonymous, and the results were kept confidential. It took about 15 min to complete the questionnaire.

The data was collected through the online questionnaires, which included three parts. Specifically, the first part was about participants' basic information including age, gender, current address, education, and so on. The second part contained 17 questions related to online health platform information, online trust, and the willingness to conduct online health consultation. The third part consisted of 4 items about health consciousness. Responses of 412 valid questionnaires were collected. Descriptive statistics of participants' characteristics were shown in Table 1.

**Table 1:** Descriptive statistics of participants' characteristics.

**Measure**	**Category**	**Frequency**	**Percentage**
			**(%)**
Gender	Male	201	48.8
	Female	211	51.2
Current address	Urban	370	89.8
	Town	28	6.8
	Rural	14	3.4
Age	25–35	290	70.4
	36–45	92	22.3
	46–55	21	5.1
	>56	9	2.2
Education	Junior high school and less	3	0.7
	Senior high school	17	4.1
	Secondary vocational school	15	3.6
	Junior college or undergraduate	349	84.7
	Postgraduate and above	28	6.8

### Measures

#### Willingness to conduct online health consultation

Willingness to conduct online health consultation was measured by modifying the items used by Wan et al. (34) and Pappas (35). The scale included three items (e.g., “If I have the chance, I am willing to consult about health problems in the online health community”). Participants rated items on a seven-point Likert scale ranging from 1 (strongly disagree) to 7 (strongly agree). In this study, Cronbach's α of the total scale was 0.72.

#### Online health platform information

The cognition-based information can be divided into two elements in this study: competence and benevolence. The former was measured by one item (i.e., “I can judge physicians' competence by knowing their clinical titles in the online health community”), and the latter was also assessed by one item (i.e., “I can judge physicians' benevolence by knowing the number of the articles which they voluntarily publish in the online health community”) (6). The affect-based information refers to online reviews, virtual gifts, and the degree of satisfaction reported by patients (6). Pavlou (20) pointed out that the institution-based information included the aspects of monitoring and accreditation. In this study, the monitoring aspect was evaluated by three items (e.g., “I can know that the online health platform assures that all consultations are conducted properly”), and the accreditation was measured by two items (e.g., “I can know that the online health platform makes a substantial effort to assess the doctors' true competencies”), which were adapted from Pavlou (20). All items are measured on a seven-point Likert scale ranging from 1 (strongly disagree) to 7 (strongly agree). The Cronbach's α for the total scale was 0.85.

#### Online trust

Online trust was measured by four items (e.g., “I think the platform of online health community is trustworthy and honest”) selected from Chen and Barnes (36). It is rated on a seven-point Likert scale, ranging from 1 (strongly disagree) to 7 (strongly agree). In this study, Cronbach's α of the scale was 0.84.

#### Health consciousness

The Health Consciousness Scale (HCS) developed by Gould (37) was used to assess participants' health consciousness. There are four items in total (e.g., “I'm very involved with my health”), each of which was rated from 0 (a statement doesn't describe you at all) to 4 (a statement describes you very well). In this study, Cronbach's α of the scale was 0.72.

### Statistical analyses

In this study, we used SPSS 26.0 to conduct common method deviation test and correlation analysis among all variables. Then we used the PROCESS macro for hypothesis testing.

## Results

### The test of common method deviation

We used SPSS 26.0 to conduct exploratory factor analysis for all items in the variables to see if Common Method Deviation was a serious problem in this study. The results showed that the eigenvalues of 5 factors were >1, and the first factor can only explain 31.26%, which was <40% critical criterion. Therefore, there was no serious problem of common method bias in the data of this study.

### Correlation analysis

The correlation matrix for all variables is demonstrated in Table 2. The results showed positive relationships among all variables (see Table 2).

**Table 2:** Correlation matrix for all study variables.

	**1**	**2**	**3**	**4**	**5**	**6**
1. Cognition-based information	1.00					
2. Affect-based information	0.500^***^	1.00				
3. Institution-based information	0.622^***^	0.515^***^	1.00			
4. Online trust	0.499^***^	0.544^***^	0.630^***^	1.00		
5. Health consciousness	0.148^**^	0.257^***^	0.290^***^	0.206^**^	1.00	
6. Willingness to conduct online health consultation	0.466^***^	0.581^***^	0.603^***^	0.697^***^	0.277^***^	1.00
Mean	5.067	5.298	5.190	5.231	4.396	5.522
SD	0.899	0.863	0.742	0.783	0.417	0.791

** Sig. < 0.01;

*** Sig. < 0.001.

### Hypothesis testing

#### Testing the mediating effect of online trust

Before using PROCESS for mediating and moderating effects, we need to examine whether the variables meet the following conditions: (1) the true relationship is linear, (2) errors are normally distributed, (3) homoscedasticity of errors, and (4) independence of the observations (38). According to the analysis of the scatter plot and normal P-p plot in SPSS, the relationships between variables in this study were linear, and the errors were normally distributed. The fitting line of a scatter plot for analysis of errors' homogeneity was parallel to the abscissa, so that the errors were homogeneous. The variance inflation factor (VIF) values of all predictive variables were <10, which indicates that the observations were independent in the study. It can be seen that the variables in this study met the four conditions mentioned above.

The PROCESS Model 4 compiled by Hays et al. (39) was used to test the mediating effect of online trust in the effect of online health platform information on patients' willingness to conduct online health consultation. Besides, age, gender, current address, and level of education were included as control variables in the entire model. The results (see Table 3) showed that cognition-based information, affect-based information, and institution-based information all significantly predicted the willingness to do online health consultation (β_cognition_ = 0.410, *p* < 0.001; β_affect_ = 0.532, *p* < 0.001; β_institution_ = 0.643, *p* < 0.001), supporting Hypothesis 1 to 3. After the mediating variable was involved in the model, cognition-based information, affect-based information, and institution-based information positively predicted online trust (β_cognition_ = 0.435, *p* < 0.001; β_affect_ = 0.493, *p* < 0.001; β_institution_ = 0.671, *p* < 0.001), and online trust positively forecasted the willingness to do online health consultation (β = 0.458, *p* < 0.001).

**Table 3:** The moderated mediating effect between online health platform information and patients' willingness to do online health consultation.

	**Outcome variable:**				**Outcome variable:**			
	**Online trust**				**Patients' willingness to do**			
					**online health consultation**			
**Variables**	**Model 1**		**Model 2**		**Model 3**		**Model 4**	
	**β**	***t***	**β**	***t***	**β**	***t***	**β**	***t***
Cognition-based information	0.435	11.66^***^	0.209	4.949^***^	0.410	10.675^***^	0.139	3.933^***^
Affect-based information	0.493	13.117^***^	0.128	3.195^**^	0.532	14.448^***^	0.263	7.206^***^
Institution-based information	0.671	21.630^***^	0.536	12.148^***^	0.643	15.299^***^	0.215	3.965^***^
Online trust							0.458	8.956^***^
Health consciousness			0.010	0.293				
Cognition-based information *health consciousness			0.266	3.283^**^				
Affect-based information *health consciousness			0.171	2.083^*^				
Institution-based information *health consciousness			0.177	2.198^*^				
*R^2^*	0.570		0.583		0.476		0.565	
*F*	76.487^***^		62.549^***^		52.518^***^		65.431^***^	

* Sig. < 0.05;

** Sig. < 0.01;

*** Sig. < 0.001. Controlling for age, gender, current address, and level of education in the entire group.

Furthermore, the Bootstrap Method indicated that online trust significantly mediated the relationships between three types of online health platform information and the willingness to do online health consultation [mediating effect_cognition_ = 0.199, 95% Boot CI = (0.145, 0.256); mediating effect_affect_ = 0.226, 95% Boot CI = (0.169, 0.287); mediating effect_institution_ = 0.307, 95% Boot CI = (0.193, 0.422)], supporting Hypothesis 4. Therefore, online trust mediated the relationship between cognition-based/affect-based/institution-based information and the willingness to do online health consultation.

#### Testing the moderated mediating effect

**Figure 2:**
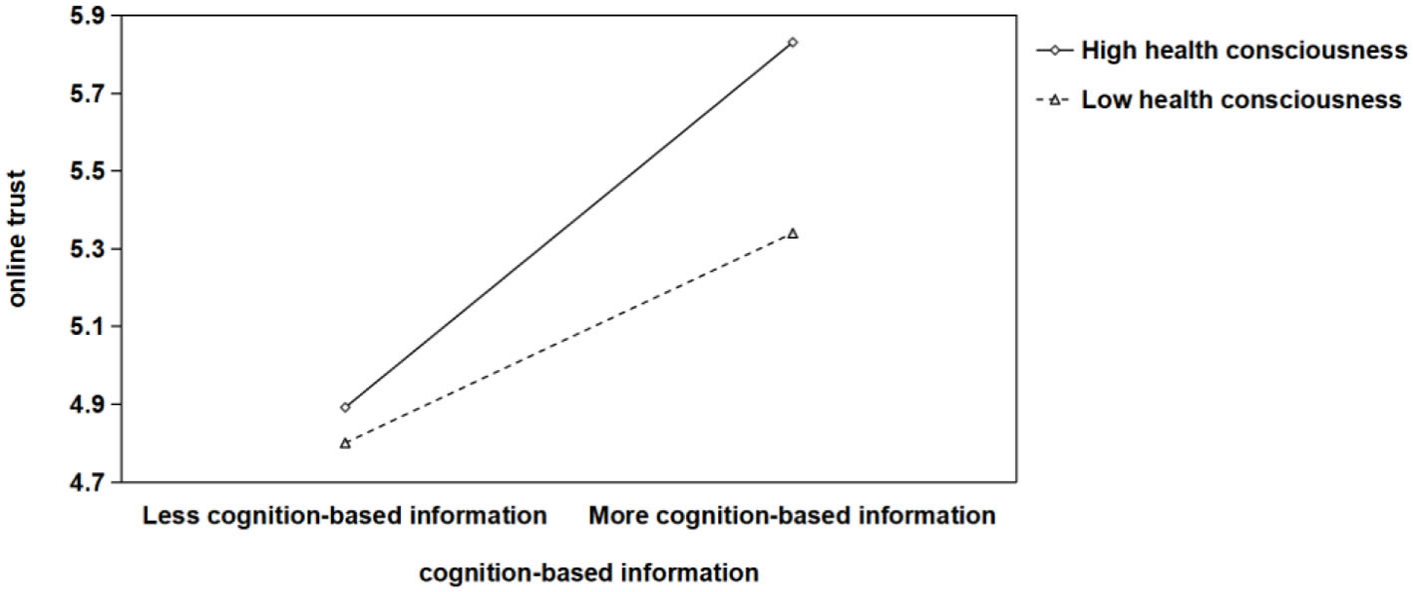
Interaction chart for different levels (±1 standard deviation) of health consciousness in the mediating model of “cognition-based information → online trust → patients' willingness to conduct online health consultation”.

**Figure 3:**
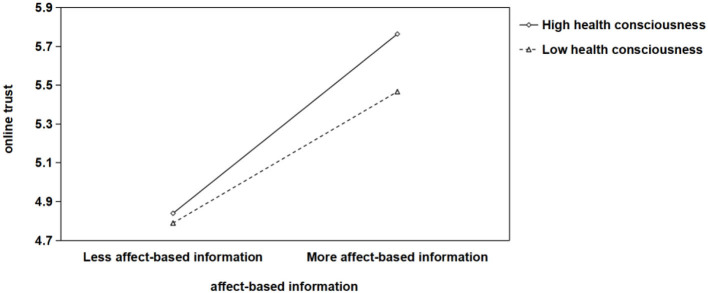
Interaction chart for different levels (±1 standard deviation) of health consciousness in the mediating model of “affect-based information → online trust → patients' willingness to conduct online health consultation”.

**Figure 4:**
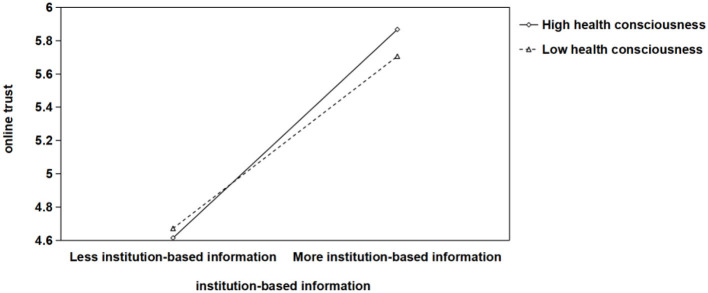
Interaction chart for different levels (±1 standard deviation) of health consciousness in the mediating model of “institution-based information → online trust → patients' willingness to conduct online health consultation”.

We further adopted PROCESS Model 7 to examine moderated mediation and conditional indirect effects, with health consciousness situated as a moderator in all paths “cognition-based/affect-based/ institution-based information → online trust → willingness to conduct online health consultation.” In addition, age, gender, current address and level of education were included as control variables in the entire model. The results revealed that the interaction terms of three types of online health platform information and online trust were positive and significant (β_cognition*online trust_ = 0.266, *p* < 0.01; β_affect*online trust_ = 0.171, *p* < 0.05; β_institution*online trust_ = 0.177, *p* < 0.05). Further, the simple slope tests were conducted at high and low levels of health consciousness, defined as one standard deviation below and above the mean. As shown in Figure 2, the positive relationship between the cognition-based information and online trust was stronger when health consciousness was high (*simple slope* = 0.522, *p* < 0.001) than when it was low (*simple slope* = 0.300, *p* < 0.001). Likewise, as shown in Figure 3, the positive relationship between the affect-based information and online trust was stronger when health consciousness was high (*simple slope* = 0.535, *p* < 0.001) than when it was low (*simple slope* = 0.392, *p* < 0.001). In addition, Figure 4 showed that the positive relationship between the institution-based information and online trust was stronger when health consciousness was high (*simple slope* = 0.845, *p* < 0.001) than when it was low (*simple slope* = 0.697, *p* < 0.001).

The indirect effect of cognition-based information on willingness to do online health consultation through online trust was stronger when health consciousness was high [indirect effect = 0.325, 95% Boot CI = (0.256, 0.398)] than when it was low [indirect effect = 0.187, 95% Boot CI = (0.121, 0.273)]. Also, the indirect effect of affect-based information on willingness to do online health consultation through online trust was stronger when health consciousness was high [indirect effect = 0.292, 95% Boot CI = (0.222, 0.368)] than when it was low [indirect effect = 0.214, 95% Boot CI = (0.146, 0.312)]. Similarly, the indirect effect of institution-based information on willingness to do online health consultation through online trust was stronger when health consciousness was high [indirect effect = 0.368, 95% Boot CI = (0.305, 0.479)] than when it was low [indirect effect = 0.287, 95% Boot CI = (0.276, 0.325)]. These findings indicated that the mediated relationship between cognition-based/affect-based/institution-based information and willingness to online health consultation through online trust was stronger with the increasing of health consciousness. As a result, Hypothesis 5 was supported.

## Discussion

The study mainly discussed how online trust and health consciousness affect the relationship between online health platform information and patients' willingness to online health consultation. The findings of this study have important theoretical contributions and practical implications.

### Theoretical contributions

This study makes several theoretical contributions to the literature in the following three aspects. First, this study fills in the gap of previous literature and extends the application of Medium System Dependency Theory into the research about online health information. On the one hand, the existing research has been lack of systematicness in the selection of information on online health platform, and only focused on single-dimensional information, such as physicians' personal quality (6), patients' online reviews (5). Our findings probed into multi-dimensional information (cognition-based, affect-based, and institution-based information) on the online health platform, and revealed their effects on patients' intention to participate in online health consultation. On the other hand, the Medium System Dependency Theory provides support for the relationship between online health platform information and the willingness to conduct online health consultation. To our knowledge, this study takes the first step to apply this theory to the willingness to do online medical consultation. Thus, this research extends the application of Medium System Dependency Theory into the area of online healthcare.

Second, the findings adds new evidences for the Web Trust Model in the area of online healthcare service. Drawing the Web Trust Model (25), cognition-based information, affect-based information and institution-based information on the online health platform can help patients build online trust in the non-face-to-face physician-patient interaction. And the building of online trust can lead to a higher willingness to do online health consultation (40, 41). The results of this research showed that online health platform information can positively influence patients' willingness to carry out online health consultation through the indirect effect of online trust. The Web Trust Model provides a solid theoretical basis for explaining the mechanism underlying the relationship between online health platform information and patients' willingness to do online health consultation. The conclusion of this study echoes the research evidence in the field of P2P sharing platform (42) and expands the application scope of the Web Trust Model.

Third, this study identified health consciousness as the boundary condition for the indirect effect of online health platform information on the willingness to conduct online health consultation through online trust. In accordance with our expectations, health consciousness moderated the mediating model of “online health platform information → online trust → willingness to do online health consultation.” That is, health consciousness can strengthen the positive relationship between online health platform information and patients' willingness to do online health consultation *via* online trust. Compared to individuals with low health consciousness, online health platform information has a more significant effect on online trust and willingness to do online healthy consultation for individuals with high health consciousness. Prior research has already identified health consciousness as an crucial variable in determining people's health service perception (30, 43). For example, Espinosa and Kadić-Maglajlić (30) have highlighted that health consciousness was an important antecedent of physician-patient trust. Likewise, Handayani et al. (43) have demonstrated that health consciousness is an important factor affecting adopting intention of mobile health application. However, little research has investigated the boundary conditions affecting individuals' acceptance of online health consultation services from the perspective of health consciousness. Echoing the above previous research, this study introduces health consciousness as a critical moderator. Therefore, this paper makes theoretical contributions by offering insights into the boundary condition under which the online health platform information could influence patients' online trust and willingness to do online health consultation.

### Practical implications

The present study also offers important implications for the online health platform in the context of digitalization transformation. First, the findings point the way to which type of information should be provided for patients. The results of this study indicated that online health platform information positively affects patients' willingness to do the online health consultation. Especially in the context of digitalization transformation, the internet is the main source of health information for people. Therefore, it is necessary for the platform to manage the content of online health platform information. The first aspect is cognition-based information that reflects physicians' personal qualities, including physicians' major areas of expertise, education background, total online response time, and so on Mao and Zhao (44). The second one is some affect-based information about physicians' online reputation reported by patients, including recommend index, efficacy satisfaction, and attitude satisfaction. The third one is institution-based information about the structured guarantees for the physician-patient interaction provided by the online health platform, including the screening and review of physicians, the protection of patients' health information and the guarantee of carrying out health consultation services. In conclusion, the online health platform should provide and update cognition-based information, affect-based information, and institution-based information for patients, thus promoting their willingness to do online health consultation with physicians.

Second, these findings suggest that the online health platform should adopt various methods to improve patients' online trust. The study indicated that online trust plays a significant mediating role in the relationship between online health platform information and the willingness to do online health consultation. In order to improve patients' willingness to carry out online health consultation, it is necessary for the online health platform to take measures to enhance patients' online trust. On the one hand, the platform should hide patients' health information and ensure that patients' health consultation services are guaranteed as much as possible. Also, the platform should offer complain channels for patients and give feedback for service disputes in time. On the other hand, managers should also pay attention to the upgrading of online health platform systems and technologies. Some questions posted by users on the online health platform haven't been answered due to the limited time of medical experts. Recently, scholars have confirmed that a new generative dialog system, named MSSGK, can effectively promote the efficiency and quality of online health consultation (45). Therefore, it's expected to build the generative dialog system for automatically returning responses. From this, patients would create a friendly relationship with online health platform and build trust in it.

Third, it is suggested that the online health platform pay more attention to patients with high health consciousness and regard them as target consumers. The results of this research uncovered that health consciousness can positively regulate the effect of online health platform information on online trust. In other words, for individuals with high health consciousness, online health platform information can trigger a higher level of online trust, and thus lead to patients' higher willingness to do online health consultation than those with low health consciousness. Accordingly, on the one hand, the platform should focus on the users who have browsed the online health platform information for many times. Platform managers can know about the users who frequently browse the online health platform information from the background data and then push relevant publicity information of the platform to them, which can nudge these users to choose online consultation services. On the other hand, the platform can provide more information beneficial to improving individuals' health consciousness and free services of physical examination to help patients learn more about their health conditions, thus further facilitating their willingness to conduct online health consultation.

### Conclusions, limitations, and future research directions

This study contributes to previous literature by investigating the potential mechanism that links multi-dimensional information on online health platform and patients' willingness to conduct online health consultation. The results revealed that cognition-based information, affect-based information and institution-based information on the online health platform positively predicted patients' willingness to conduct online health consultation. Meanwhile, online trust mediated the relationships between online health platform information and patients' willingness to carry out online health consultation. Furthermore, health consciousness significantly moderated the positive effect of online health platform information on online trust, that is, this relationship was stronger under high vs. low health consciousness.

Although this study has important theoretical contributions and practical implications, some limitations in this study should also be acknowledged. First, this study only examined the effects of three types of information on online health platform on patients' willingness to carry out online health consultation. However, there may be other aspects or characteristics of information on the platform that influence patient' willingness to do online health consultation. And the possible association between these factors and patient' willingness to do online health consultation are worth further investigation in future research. Second, the cross-sectional design was adopted in this study, thus it was difficult to carry out the causal inference. In the future, researchers can adopt longitudinal research designs or field experiment to examine the effect of online health platform information on patients' willingness to carry out online health consultation and its inner mechanisms. Third, linear models were used for analysis in this study, which couldn't capture the whole situation of statistical relationships among variables and had some deviation (46). Future research can use more diversified data analysis methods, such as structural equation model, to solve this problem.

## Data availability statement

The original contributions presented in the study are included in the article/supplementary material, further inquiries can be directed to the corresponding author.

## Ethics statement

The studies involving human participants were reviewed and approved by the Institutional Review Board of Xuzhou Vocational College of Industrial Technology. The patients/participants provided their written informed consent to participate in this study.

## Author contributions

YX, ZY, and HJ prepared the study concept and design. YX and ZY wrote the main manuscript text and analyzed the data. HJ and PS reviewed and edited the draft. YX and HJ provided funds to conduct the study. All authors have read and agreed to the published version of the manuscript.

## Funding

This study was supported by the National Natural Science Foundation of China (72072172 and 71672187), Social Science Foundation of Jiangsu Province (20GLB005), the Key Projects of the 13th Five-Year Plan for Education Science of Jiangsu Province (B-a/2020/01/11), the Fundamental Research Funds for the Central Universities (2021ZDPYYQ006), and the Ph.D. project of Xuzhou Vocational College of Industrial Technology (XGY2020EB01).

## Conflict of interest

The authors declare that the research was conducted in the absence of any commercial or financial relationships that could be construed as a potential conflict of interest.

## Publisher's note

All claims expressed in this article are solely those of the authors and do not necessarily represent those of their affiliated organizations, or those of the publisher, the editors and the reviewers. Any product that may be evaluated in this article, or claim that may be made by its manufacturer, is not guaranteed or endorsed by the publisher.
